# Medical Items

**Published:** 1897-05

**Authors:** 


### *MEDICAL ITEMS.*

---

DR. R. E. GRIGGS, of Columbus, Ga., died April 18th.

---

DR. O. A. SIMS, of Norcross, died April 30th, aged 28.

---

THE College of Physicians and Surgeons, Chicago, has become the Medical Department of the University of Illinois.

---

THE Editorial Department of the *Universal Medical Annual* (Sajous') will be transferred from Paris to Philadelphia this month.

---

Hepatic doctors now are seen no more,
The hunt for bile has long been given o'er;
Whoever would now a reputation make
Deserts the bile—the bug to overtake. —*Practical Medicine.*

---

THE first negro woman to obtain the medical degree is Emma Wakefield, of Louisiana. She graduated in the Medical Department of the New Orleans Colored University, and lately was licensed to practice by the Louisiana Board of Examiners.

---

THE Mayor of Savannah has appointed Dr. J. H. Graham Health and Quarantine Officer for the unexpired term of Dr. W. F. Brunner, lately resigned. Dr. Brunner has accepted the post of Sanitary Inspector at Havana, under the Marine Hospital service.

---

DR. EUGENE R. CORSON, of Savannah, favors us with an exceedingly instructive and interesting monograph entitled "Some Clinical Jottings on Five Hundred Cases of Labor." The author has kept careful notes of his labor cases and has here analyzed them in a manner to convey important lessons to the reader.

DRS. JNO. M. KEATING and Henry C. Coe are completing a voluminous work on clinical gynecology, to be issued from the Lippincott press of Philadelphia.—*Am. Med. Journalist.*

As one of the above named gentlemen has been dead more than two years, one wonders where the editor of the *American Medical Journalist* has been confined in the meantime.

---

THE Medical Association of Alabama, in session in Selma, April 21–23, elected following officers for ensuing year: President, Dr. L. L. Hill, of Montgomery; 1st Vice-President, Dr. J. C. LeGrand, of Anniston; 2d Vice-President, Dr. E. L. Marechel, of Mobile; Councillors, Drs. Seeley, of Montgomery, Saunders, of Mobile, and Furniss, of Selma; Orator, Dr. W. W. Harper, of Selma. The Association will meet next year in Birmingham.

---

PHYSICIANS who contemplate attending the International Medical Congress at Moscow, August 18th to 26th, will do well to write to the Tourist Agents, Thos. Cook & Son, 26 Broadway, New York, or 234 S. Clark St., Chicago. Organized parties of physicians will be conducted by these agents under the charge of traveling directors, who are thoroughly familiar with the languages and customs of the countries visited. The parties will leave New York July 3d, and spend several weeks in travel before the opening of the Congress. The tours are planned for about ninety days.

---

AN ardent sanitarist is a member of the Texas Legislature this year, and he has prepared a bill which he thinks will greatly benefit his fellow-citizens. The bill, if passed, will revolutionize the marriage system of the State. The intending groom must previously undergo a thorough physical examination at the hands of a competent medical practitioner in good standing, and be possessed of said physician's sworn certificate of physical soundness. The prospective bride must also have undergone a similar ordeal, and a like certificate in her behalf must be submitted. Not only this, but both parties "to the contract" must file sworn statements attesting the fact that neither of them are subject in a hereditary way to any disease that might in like manner transmit tendencies thereto in their probable offspring. The county clerk must then satisfy himself that these "credentials" are perfect before granting the license to marry.—*Jour. Am. Med. Association.*

---

THE Board of Trustees of Jefferson Medical College, Philadelphia, has taken another forward step in adopting plans for a special spring course of instruction in the new laboratories of the college, and in making elaborate preparations for summer laboratory courses and for post-graduate teaching. The arrangements are that post-graduate instruction in the various sub-departments of pathology shall be open to holders of medical degrees only; that undergraduate instruction shall be given between May 1 and October 1 of each year, such instruction to be credited on next year's work; and that any student or graduate of the college who may show special ability may be appointed by the Professor of Pathology to do prize work in the laboratories, or work which shall be honored by publication as original investigation. In this way, without at all impairing the efficiency or restricting the scope of its regular winter course, the Jefferson College establishes a complete system of summer laboratory work. The new departure taken by the progressive management of Jefferson College will be widely appreciated and heartily approved.

---

THE Seventh Annual Meeting of the National Confederation of State Medical Examining and Licensing Boards will be held in the small banquet hall of the Hotel Walton, at Philadelphia, Monday, May 31, 1897, at ten o'clock A.M. An interesting program has been arranged. The object of the confederation is to consider questions pertaining to State control in medicine and to compare methods in vogue in the several States; the collection and dissemination of information relating to medical education, and to consider propositions that have for their purpose advancement of the standards in the United States. A cordial invitation is extended to all members and ex-members of State medical examining boards, and to physicians, sanitarians, and educators who are friendly to the objects named, to attend the meeting and participate in its proceedings. Officers: President, William W. Potter, New York; Vice-Presidents, Charles A. L. Reed, Ohio, and J. N. McCormick, Kentucky; Secretary and Treasurer, A. Walter Suiter, Herkimer, N. Y.

---

AN important event in New York medical circles is the consolidation of the University Medical College with Bellevue, under the joint name of The New York University Bellevue Medical College. Every one, we are sure, will approve of the union. We believe it is in the interest of a higher medical education, and it gives prominence to the idea that the cause of higher medical education is to be advanced, not in the organization of more schools, but in the suspension of some already existing and in the improvement of others. Consolidation of colleges is another means to this end. The new school above named will be under the control of the New York University, the faculty of each, we understand, having resigned so as to give the Trustees of the University absolute freedom in the construction of a new faculty. The college will have a liberal endowment, the facilities for medical teaching will be made equal to the best anywhere, and no pains or expense will be spared to make the new college occupy the very front rank among thoroughly equipped and up-to-date medical colleges.

